# Bredemolic Acid Ameliorates Selected Liver Function Biomarkers in a Diet-Induced Prediabetic Rat Model

**DOI:** 10.1155/2020/2475301

**Published:** 2020-02-20

**Authors:** Akinjide Moses Akinnuga, Angezwa Siboto, Bongiwe Khumalo, Ntethelelo Hopewell Sibiya, Phikelelani Ngubane, Andile Khathi

**Affiliations:** ^1^Department of Physiology, School of Laboratory Medicine and Medical Sciences, College of Health Sciences, University of KwaZulu-Nata, Westville, Durban, South Africa; ^2^Department of Pharmacy and Pharmacology, Rhodes University, Grahamstown, South Africa

## Abstract

**Background:**

Prediabetes is an intermediary hyperglycaemic state that precedes type 2 diabetes mellitus (T2DM) in which abnormal metabolism of glucose and lipids occurs in organs such as the liver. Evidence has shown that, about 70% of T2DM patients develop hepatic dysfunction which is found to begin during the prediabetic stage. Bredemolic acid, a pentacyclic triterpene, has been found to improve insulin sensitivity in diet-induced prediabetic rats. The effects of this compound on liver function, however, are unknown. This study was therefore designed to investigate the effects of BA on liver function in high fat-high carbohydrate (HFHC) diet-induced prediabetic rats.

**Methods:**

Thirty-six (36) male rats that weigh 150 g–180 g were divided into two groups, the non-prediabetic (*n* = 6) and the prediabetic groups (*n* = 6) and the prediabetic groups (*n* = 6) and the prediabetic groups (

**Results:**

The induction of prediabetes resulted in increased release of liver enzymes (AST and ALT), increased liver glycogen and triglyceride, lipid peroxidation, and decreased sterol regulatory element-binding protein (SREBP1c) and antioxidant enzymes. However, the administration of BA decreased liver enzyme concentrations, decreased hepatic oxidative stress, and improved antioxidant enzymes such as SOD and GPx.

**Conclusion:**

BA administration improved liver function in diet-induced prediabetic rats in the presence or absence of dietary intervention.

## 1. Introduction

Prediabetes is a state of intermediate hyperglycaemia that causes abnormal changes in intracellular metabolism of most body tissues including the liver [1]. Presently, the observed increase in the prevalence of prediabetes and type 2 diabetes mellitus (T2DM) in developed and developing countries is reported to be due to sedentary lifestyles coupled with high-caloric diets [1–3]. However, studies have shown that excessive intake of high-caloric diets induces skeletal muscle insulin resistance which results into the shunting of glucose from the skeletal muscle to the liver thereby leading to increased hepatic glycogen production and storage [4–6]. Several studies have shown that continuous intake of high quantities of fats and carbohydrates alters liver function by accumulation of ectopic fats as a result of *de novo* lipogenesis which is mediated by transcription factors such as sterol regulatory element-binding protein (SREBP1c) under insulin action [7, 8]. Moreover, excessive hepatic accumulation of free fatty acid or triglyceride leads to hepatic insulin resistance, hepatic dysfunction, and nonalcoholic fatty liver disease (NAFLD) that is characterized by fat infiltration into the hepatocytes [9–14]. Consequently, the infiltration of fat into the hepatocytes triggers oxidative stress, and reduces antioxidant enzymes production and caused an inflammatory cascade of reactions that produce progressive fibrotic hepatic damage known as nonalcoholic steatohepatitis (NASH). Cross-sectional studies have demonstrated that liver function markers such as alanine aminotransferase (ALT) and aspartate aminotransferase (AST) are altered due to oxidative stress and hepatic dysfunction [15–18]. However, it has been established that approximately 70% of T2DM patients have liver dysfunction and complications [19–21]. There is also evidence from other studies that suggested that liver dysfunction and complications can also begin during the prediabetic stage [21–23].

Current treatment focuses on a combination of dietary and pharmacological interventions, but there has been reports of low compliance as patients merely use pharmacological intervention without diet modification thus reducing the efficacy of the pharmacological intervention [24–27]. Therefore, novel compounds that can ameliorate liver dysfunction in the prediabetic condition even in the absence of dietary intervention are necessary. Oleanolic acid and maslinic acid are pentacyclic triterpenes that have been found to have antidiabetic and antioxidant properties [28–30]. In our laboratory, we have shown that chronic ingestion of a high fat-high carbohydrate diet leads to the development of prediabetes which is accompanied by liver complications. We have further shown that bredemolic acid (BA), a structural isomer of maslinic acid, is able to restore glucose homeostasis in diet-induced prediabetes by improving insulin sensitivity both in presence and absence of dietary intervention [31]. However, the effects of BA on liver function in diet-induced prediabetes have not been established. Hence, the aim of this study is to investigate the effects of bredemolic acid on selected biomarkers of liver function in a diet-induced prediabetic rat model.

## 2. Materials and Methods

### 2.1. Animals

Thirty-six (36) male Sprague Dawley rats (150–180 g) obtained from Biomedical Research Unit, University of KwaZulu-Natal (UKZN), were kept under standard environmental conditions i.e., constant humidity (55 ± 5%), temperature (22 ± 2°C), 12 h day : 12 h night cycle. The animals were acclimatized for 2 weeks and consumed standard rat chow (Meadow Feeds, South Africa) and water *ad libitum* before being fed on the experimental high fat-high carbohydrate (HFHC) diet (AVI Products (Pty) Ltd., Waterfall, South Africa) to induce prediabetes. The HFHC diet consists of carbohydrate (55% kcal/g), fats (30% kcal/g), and proteins (15% kcal/g) as described in our previous study [27, 31]. All the experimental designs and procedures were carried out according to the ethics and guidelines of the Animal Research Ethics Committee (AREC) of the UKZN, Durban, South Africa.

### 2.2. Experimental Design

After acclimatization, the animals were divided into two groups, the normal diet (ND) non-prediabetic control (*n* = 6) and the HFHC diet prediabetic groups (*n* = 30). All the animals in the prediabetic group consumed HFHC diet and drinking water that was supplemented with 15% fructose for 20 weeks to induce prediabetes while the non-prediabetic control group (NPD, Group 1) fed on ND and water *ad libitum* for 20 weeks as well. At the 20^th^ week, prediabetes was confirmed by fasting blood glucose and oral glucose tolerance test which have been described in the previous research study [31].

### 2.3. Treatment of Prediabetic Animals

After 20 weeks of prediabetes induction, the non-prediabetic control (NPD, Group 1) animals were continuously fed on standard rat chow for 12 weeks. Thirty (30) prediabetic animals were randomly assigned into 5 different groups (Group 2 to Group 6, *n* = 6). Group 2 (PD) served as the untreated prediabetic control group and continuously consumed the HFHC diet for 12 weeks; Group 3 (ND + MET) were prediabetic animals that switched to standard rat chow and received metformin (MET) for 12 weeks; Group 4 (HFHC + MET) were prediabetic animals that continuously consumed HFHC diet with MET treatment; Group 5 (ND + BA) were prediabetic animals that switched to standard rat chow and received BA for 12 weeks; and Group 6 (HFHC + BA) were prediabetic animals that continuously consumed HFHC diet and received BA as treatment for 12 weeks. Treatment via oral administration of MET (7.2 mg/kg, extrapolated from 500 mg/70 kg human dose) or BA (80 mg/kg) was carried out every third day for 12 weeks as described in our previous study [31].

### 2.4. Blood Collection and Tissue Harvesting

After the 12^th^ week treatment period, the animals were sacrificed. The animals were placed in a gas anaesthetic chamber (Biomedical Research Unit, UKZN, Durban, South Africa) and anaesthetised with Isofor (100 mg/kg, Safeline Pharmaceuticals, Roodepoort, South Africa) for 3 minutes. Blood samples were collected from the animals using cardiac puncture and put into different precooled EDTA containers. The blood samples were centrifuged (Eppendorf centrifuge 5403, Germany) at 4°C, 503*g* for 15 minutes to obtain plasma. Each of the plasma was aspirated into plain sample bottles and stored at −80°C in a BioUltra freezer (Snijers Scientific, Tilburg, Holland) until ready for biochemical analysis. Also, the liver tissue samples were excised, weighed, and rinsed in cold normal saline solution and snapped frozen in liquid nitrogen before storage in the BioUltra freezer for biochemical analysis of selected metabolic parameters.

### 2.5. Relative Liver Weight

The relative liver weights of all the animals in each experimental group were determined from the percentage of the ratio of liver weight to the body weight i.e.,

(1) $$\begin{array}{c} \text{relative liver weight}=\frac{\text{liver weight}}{\text{body weight}}\times 100. \end{array}$$

### 2.6. Biochemical Analysis

Liver enzymes (AST and ALT) in the plasma were analysed with IDEXX Catalyst One Chemistry Analyzer (IDEXX Laboratories Inc., Westbrook, USA) while SREBP1c in the liver homogenate was analysed by following specific ELISA kit procedures using manufacturer's instructions (Elabscience Biotechnology Co., Ltd., Houston, TX, USA). Fasting blood insulin (FBI) was also analysed and determined as reported in our previous study [31].

### 2.7. Liver Triglycerides

The preparation of liver tissue samples and the homogenate medium used for determination of hepatic triglyceride were according to the manufacturer instruction in the triglyceride assay kit (Elabscience Biotechnology Co., Ltd., Houston, TX, USA). 50 mg of liver tissue was homogenized on ice in 500 *μ*l phosphate buffer saline (PBS) and centrifuged at 8000 rpm for 10 minutes, 4°C. The supernatant was then aspirated into Eppendorf tubes, and triglycerides were determined using the triglyceride assay kit as instructed in the manufacturer's manual. The absorbance of the samples was measured at 510 nm by using Spectrostar Nanospectrophotometer (BMG Labtech, Ortenburg, LGBW Germany).

### 2.8. Liver Glycogen Assay

Glycogen assay was determined in the liver by following previous established protocol [27, 28, 32]. The absorbance was determined by using the Spectrostar Nanospectrophotometer at 620 nm.

### 2.9. Lipid Peroxidation and Antioxidant Profile

The concentration of malondialdehyde (MDA) in the liver was determined to estimate the amount of lipid peroxidation according to previously described protocol [29, 32]. Furthermore, the antioxidant profile of the liver was determined by measuring the activities of superoxide dismutase (SOD) and glutathione peroxidase (GPx) according to the manufacturer's instructions (Elabscience Biotechnology Co., Ltd., Houston, TX, USA).

### 2.10. Statistical Analysis

The statistical data were presented in mean ± SEM. The data were analysed by two-way analysis of variance (ANOVA) with Bonferroni test (post hoc test) via GraphPad Prism 5 software. Also, Pearson's correlation was used to calculate the correlation coefficient between FBI and hepatic SREBP1c through the GraphPad Prism 5. The level of statistical significance was determined at *p* < 0.05.

## 3. Results

### 3.1. Relative Liver Weight

**Figure 1:**
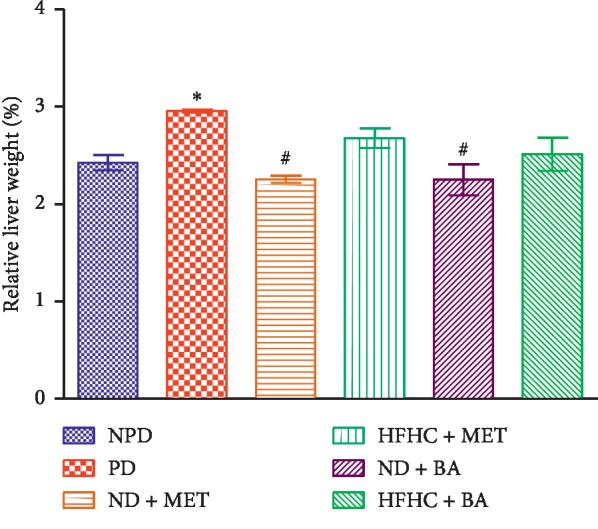
Effects of BA with the presence or absence of dietary intervention on the relative liver weight in prediabetic rats. ^*∗*^*p* < 0.05 in comparison with NPD; ^#^*p* < 0.001 in comparison with PD.

The effects of BA treatment on relative liver weights in non-prediabetic and prediabetic rats with or without diet intervention were determined. The relative liver weight of untreated prediabetic (PD) rats was significantly increased by comparison with the non-prediabetic control (NPD) rats (*p* < 0.05). However, the relative liver weight of the animals is dependent on the type of diet administered. Therefore, the administration of BA or MET and diet intervention significantly decreased the relative liver weight when compared with PD (*p* < 0.05), see Figure 1.

### 3.2. Liver Enzymes

**Figure 2:**
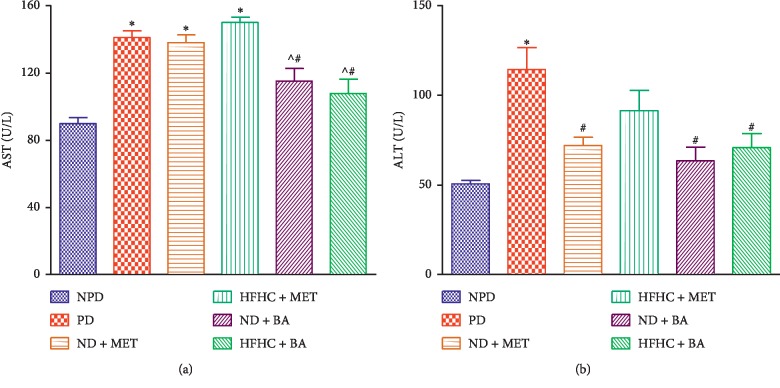
Effects of BA with the presence or absence of dietary intervention on the plasma AST and ALT in prediabetic rats. ^*∗*^*p* < 0.001 in comparison with NPD; ^#^*p* < 0.05 in comparison with PD.

Plasma AST and ALT concentrations in the PD group were significantly increased (*p* < 0.01) compared with the NPD group. However, the administration of BA with or without diet intervention significantly decreased the plasma AST and ALT concentrations when compared with PD. The plasma ALT levels of metformin-treated rats with diet intervention (ND + MET) were significantly decreased when compared with PD while the plasma AST of ND + MET was insignificantly different when compared with PD (*p* < 0.05), see Figure 2.

### 3.3. SREBP1c

**Figure 3:**
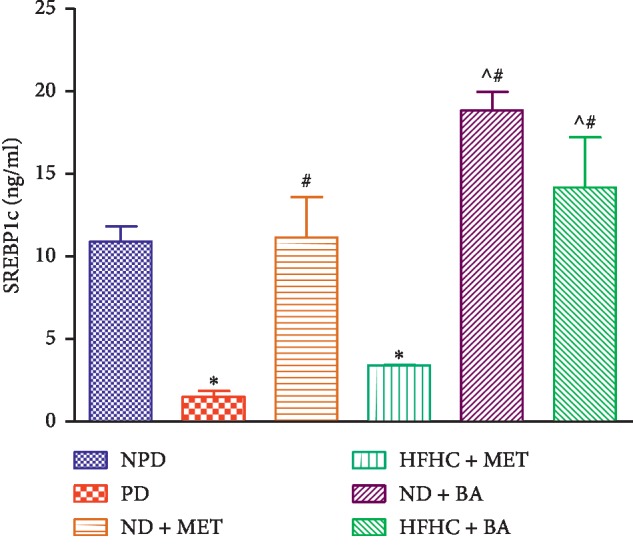
Effects of BA with the presence or absence of dietary intervention on the liver SREBP1c in prediabetic rats. ^*∗*^*p* < 0.001 in comparison with NPD, ^#^*p* < 0.001 in comparison with PD, and *^*p < 0.01 in comparison with HFHC + MET.

The liver SREBP1c concentration was determined in non-prediabetic and prediabetic rats. The liver SREBP1c levels were significantly decreased in PD groups when compared with the NPD group (*p* < 0.001). The administration of BA with or without diet intervention significantly increased the liver SREBP1c concentration in comparison with the PD group (*p* < 0.001). Interestingly, the administration of metformin with diet intervention (ND + MET) significantly increased the SREBP1c concentration when compared with the PD group (*p* < 0.05). The administration of metformin in the absence of dietary intervention did not have any significant effects when compared with the PD control, see Figure 3.

### 3.4. FBI and Hepatic SREBP1c Correlation

The correlation between FBI and hepatic SREBP1c was determined in all the groups under different experimental conditions as indicated in Table 1. There was a significant negative correlation between FBI and hepatic SREBP1c in PD, HFHC + MET, and ND + MET groups (*r* = −0.9144, −0.8869, and −0.8691, respectively) at *p* < 0.05. Therefore, as FBI increased significantly in impaired insulin signaling, there was significant decrease in hepatic SREBP1c concentration. However, there was insignificant correlation between the FBI and hepatic SREBP1c in non-prediabetic (NPD) and prediabetic rats treated with BA in the absence or presence of dietary intervention.

### 3.5. Liver Triglycerides

**Figure 4:**
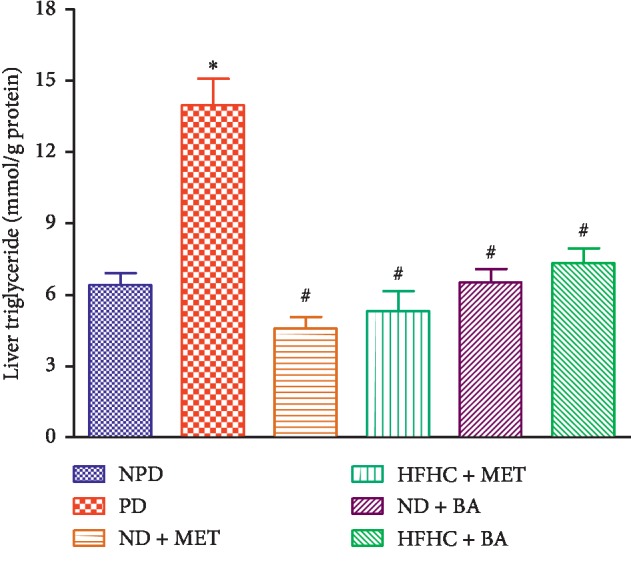
Effects of BA with the presence or absence of dietary intervention on the liver triglyceride in prediabetic rats. ^*∗*^*p* < 0.001 in comparison with NPD; ^#^*p* < 0.001 in comparison with PD.

Liver triglyceride concentrations were significantly increased in the PD group by comparison with the NPD group (*p* < 0.001). The liver triglyceride concentration of BA-treated rats with or without diet intervention significantly decreased when compared with the PD group (*p* < 0.001). Similar results were observed with the use of metformin, see Figure 4.

### 3.6. Liver Glycogen

**Figure 5:**
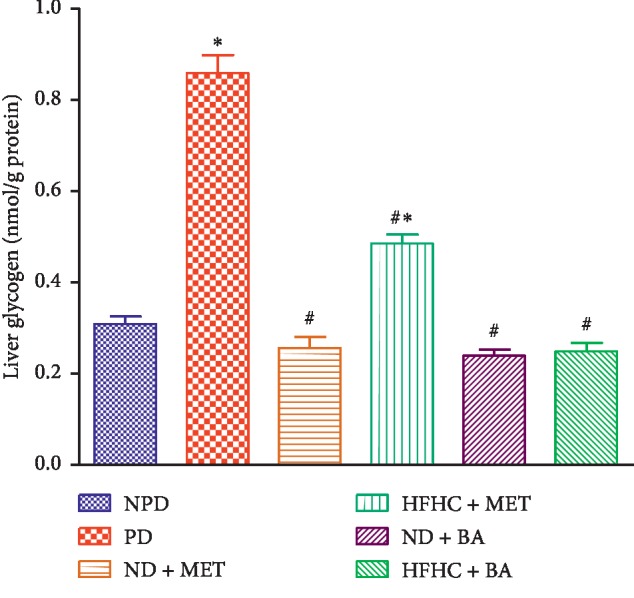
Effects of BA with the presence or absence of dietary intervention on the liver glycogen in prediabetic rats. ^*∗*^*p* < 0.001 in comparison with NPD; ^#^*p* < 0.001 in comparison with PD.

Liver glycogen concentrations of the PD group were significantly increased by comparison with the NPD group (*p* < 0.001). The administration of BA with or without diet intervention significantly decreased liver glycogen concentrations by comparison with PD (*p* < 0.001). Similarly, the administration of metformin treated with or without diet intervention significantly decreased the liver glycogen concentration when compared with PD, see Figure 5.

### 3.7. Lipid Peroxidation and Antioxidant Enzyme Activity

As shown in Table 2, liver MDA concentration in the untreated PD group was significantly increased by comparison with the NPD group (*p* < 0.001). The administration of BA and metformin with or without diet intervention significantly decreased the liver MDA concentration when compared with the PD group (*p* < 0.05). Liver SOD and GPx activities of the untreated PD group were significantly decreased when compared with the NPD group (*p* < 0.05). The SOD and GPx activities in the liver of BA-treated rats with or without diet intervention were significantly increased in comparison with those in PD group (*p* < 0.05).

## 4. Discussion

This study examined the effects of BA on selected markers of liver function in diet-induced prediabetic rats. Triterpenes such as maslinic acid and oleanolic acid have been reported to ameliorate oxidative stress in the liver via increased release of antioxidant enzymes and improved liver function via increased activity of glycogenic enzymes to decrease hepatic glucose production in diabetic rats [29, 32]. In a previous study, BA was shown to improve insulin sensitivity in the skeletal muscle by increasing the expression of GLUT 4; however, the effects of this triterpene on liver function in the prediabetic state were not determined [31]. Hence, this study is a continuation of the previous study [31] and sought to evaluate the effects of BA on selected markers of liver function in a diet-induced prediabetic rat model. The liver plays a key role in maintaining glucose homeostasis as it balances the production of glucose and the conversion of glucose to glycogen [33]. In a postprandial state, blood glucose increases, and insulin is secreted to enhance glycogenesis and inhibit glycogenolysis [34]. However, studies have shown that chronic consumption of high fat-high carbohydrate diet results in the induction of prediabetes which is characterized by hyperinsulinaemia, impaired glucose tolerance, and peripheral and hepatic insulin resistance, as well as liver damage [1, 35, 36]. In the prediabetic state, due to hyperinsulinaemia and selective muscle insulin resistance, most ingested glucose is shunted to the liver leading to increased hepatic glycogenesis [6, 37]. In addition, since the liver is insulin-independent, excess glucose in the blood can diffuse into the hepatic cells through facilitated diffusion which is mediated by glucose transporter 2 (GLUT (2)) [14, 34, 38]. Similarly, the elevated liver glycogen concentration observed in untreated prediabetic rats in this study can be attributed to the increased diversion of excess glucose to the liver. This showed that consumption of high fat-high carbohydrate diet can result into diversion of glucose to the liver as a compensatory mechanism in the presence of selective muscle resistance in the prediabetic state [34]. However, the administration of BA with or without diet intervention significantly reduced liver glycogen concentrations. Previous studies have shown that administration of BA in the prediabetic state improves insulin sensitivity in the skeletal muscle through increased GLUT 4 expression [31]. We suggest that this improved insulin sensitivity in the periphery leads to decreased amounts of glucose being shunted to the liver thus resulting in the observed decrease in liver glycogen concentrations.

In nondiabetic subjects, metabolism of glucose is largely carried out in the skeletal muscle [39, 40]. In the prediabetic state, as glucose delivery to the liver increases, *de novo* lipogenesis and hepatic lipid accumulation increase under the influence of transcription factors such as SREBP1c [6, 14, 37, 40]. SREBP1c is a major transcription factor which regulates *de novo* lipogenesis through direct activation from AKT (protein kinase B) in the insulin signaling pathway [8, 41, 42]. In the prediabetic state, when insulin signaling is impaired, the direct activation of SREBP1c by AKT is altered, and the SREBP1c expression decreases [6–8]. On the contrary, the hepatic *de novo* lipogenesis is not solely dependent on insulin signaling through activation of SREBP1c, but the activation of SREBP1c to stimulate *de novo* lipogenesis depends on insulin signaling [6, 43]. However, when the insulin signaling pathway is impaired in prediabetes, *de novo* lipogenesis is still elevated due to the substrate push mechanism in which there is increased substrate delivery to the liver followed by increased esterification of fatty acids into triglycerides [6]. In this study, we observed that the concentration of SREBP1c in the liver was significantly lowered in untreated prediabetic rats by comparison with the non-prediabetic rats. According to our correlation analysis between fasting blood insulin and hepatic SREBP1c, the decreased hepatic SREBP1c in untreated prediabetic rats may be due to the alteration of insulin signaling in the prediabetic state since SREBP1c expression is insulin-dependent. In addition, the correlation analysis showed that there was an inverse relationship between the increased fasting blood insulin and the hepatic SREBP1c concentration under the insulin-resistant condition. This observation is in correlation with previous studies which reported that insulin signaling is not totally required for hepatic lipogenesis, and that availability of the substrate can facilitate delivery of substrates into the liver for lipogenesis [6, 44]. Of note, the BA-treated rats had a significantly increased SREBP1c thus suggesting that BA ameliorated insulin signaling which may have resulted into the increased SREBP1c concentration in the liver. Furthermore, high fructose consumption has been reported to increase hepatic lipogenesis and glycogenesis [1]. Fructose, unlike glucose, is solely metabolized in the liver thereby providing additional substrates for *de novo* lipogenesis and ectopic fat accumulation in the liver, thus leading to NAFLD [1, 10]. In this study, we observed that the liver triglyceride in untreated prediabetic rats significantly increased when compared with non-prediabetic rats. The increased liver triglyceride in untreated prediabetic rats can be attributed to increased substrate delivery to the liver or decreased hepatocellular triglyceride disposal, as well as decreased fatty acid oxidation [45]. However, the administration of BA significantly decreased hepatic triglycerides, and this suggests that BA may decrease substrate delivery to the liver by divergence of the substrates to other organs for metabolism, increased *β* oxidation of fat, or increased triglyceride disposal via very low-density lipoprotein (VLDL) exportation from the liver.

Moreover, due to the increased hepatic lipogenesis and glycogenesis, the production of free radicals is elevated, and this results into oxidative stress [46]. Oxidative stress is due to an imbalance between oxidant and antioxidant enzymes [46]. Antioxidants are stable molecules that donate electrons to rampaging free radicals in order to neutralise the free radical capacity to damage tissues or organs [47, 48]. In this study, we observed that lipid peroxidation (MDA) in the liver was significantly increased, and antioxidant enzyme (SOD and GPx) production in the liver was significantly decreased in the untreated prediabetic rats when compared with non-prediabetic rats. The increased lipid peroxidation was due to increased production of free radicals while the decreased antioxidant capacity of the liver was as a result of decreased production of antioxidant enzymes (SOD and GPx) in the mitochondria of hepatocytes during prediabetes. On the contrary, BA administration with or without diet intervention significantly lowered lipid peroxidation and significantly increased the liver antioxidant enzymes. This may be due to the fact that BA neutralises the free radicals in the mitochondria of hepatocytes by donation of electron through hydroxyl radical scavenging activity which has been reported in other triterpenes [49]. This is in line with similar observations made on earlier studies using other triterpenes [28, 32, 49].

Furthermore, studies have shown that elevated liver enzymes (AST and ALT) in the plasma can be due to necrosis of the hepatocyte during liver damage [18]. AST and ALT are released into the blood stream whenever hepatocytes are damaged, and this has been reported to occur during prediabetes [18]. In this study, these enzymes were significantly elevated in untreated prediabetic rats by comparison with non-prediabetic rats. The increased liver enzymes in the plasma suggested that liver cells are damaged through oxidative stress and increased hepatic lipogenesis or glycogenesis. However, BA administration caused a decrease in the concentration of liver enzymes suggesting that BA may improve hepatic function via its antioxidant and antilipidemic effects in the liver as observed in this study. Of note, triterpenes are nontoxic antioxidants and have low pharmacokinetics of three days; therefore, the ameliorative effects of BA in the absence of dietary intervention on liver function markers compared with metformin in this study may be attributed to this low pharmacokinetic feature. In conclusion, the administration of BA in both the presence and absence of dietary modification can potentially be one of the therapeutic approaches to attenuate hepatic dysfunction or improve hepatic functions in the prediabetic state.

## Figures and Tables

**Table 1:** Correlation between fasting blood insulin (FBI) and hepatic sterol regulatory element-binding protein (SREBP1c) in non-prediabetic (NPD) rats, prediabetic control (PD), and prediabetic rats treated with BA in the presence or absence of dietary intervention. *r* = Pearson's correlation coefficient, *R*^2^ = coefficient of determination, and *n* = sample size.

Groups	Correlation analysis	Independent variable: FBI	Dependent variable: hepatic SREBP1c
NPD	*r*	0.8068	0.8068
	*R* ^2^	0.6510	0.6510
	*n*	6	6
	*p* value	0.0524^NS^	0.0524^NS^
PD	*r*	−0.9144	−0.9144
	*R* ^2^	0.8361	0.8361
	*n*	6	6
	*p* value	0.0107^*∗*^	0.0107^*∗*^
ND + MET	*r*	−0.8691	−0.8691
	*R* ^2^	0.7552	0.7552
	*n*	6	6
	*p* value	0.0246^*∗*^	0.0246^*∗*^
HFHC + MET	*r*	−0.8869	−0.8869
	*R* ^2^	0.7866	0.7866
	*n*	6	6
	*p* value	0.0185^*∗*^	0.0185^*∗*^
ND + BA	*r*	0.4651	0.4651
	*R* ^2^	0.2164	0.2164
	*n*	6	6
	*p* value	0.3526^NS^	0.3526^NS^
HFHC + BA	*r*	−0.7381	0.7381
	*R* ^2^	0.5448	0.5448
	*n*	6	6
	*p* value	0.0939^NS^	0.0939^NS^

^NS^Not significant; ^*∗*^*p* < 0.05.

**Table 2:** Effects of BA with the presence or absence of dietary intervention on the liver lipid peroxidation and antioxidant enzyme activities in prediabetic rats. Values are presented as mean ± SEM (*n* = 6).

Groups	Malondialdehyde (MDA) (nmol/g protein)	Superoxide dismutase (SOD) (nmol·min^−1^·mL·mg^−1^protein)	Glutathione peroxidase (GPx) (nmol·min^−1^·mL·mg^−1^protein)
NPD	4.11 ± 0.51	2.99 ± 0.06	1.67 ± 0.09
PD	12.34 ± 1.31^*∗*^	1.66 ± 0.22^*∗*^	1.08 ± 0.06^*∗*^
ND + MET	5.00 ± 0.26^#^	2.14 ± 0.02^#^	1.79 ± 0.07^#∧^
HFHC + MET	6.41 ± 0.27^#^	1.83 ± 0.13^*∗*^	1.05 ± 0.05^*∗*^
ND + BA	4.89 ± 0.44^#^	2.47 ± 0.06^#^	1.87 ± 0.10^#∧^
HFHC + BA	6.68 ± 0.65^#^	2.59 ± 0.02^#^	1.89 ± 0.04^#∧^

^*∗*^ *p* < 0.05 in comparison with the non-prediabetic (NPD) control, ^#^*p* < 0.05 in comparison with the prediabetic (PD) control, and ^∧^*p* < 0.05 in comparison with the HFHC + MET group.

## Data Availability

The data used to support the findings of this study are available upon request from the corresponding author. However, the data on body weight, fasting blood insulin (FBI), fasting blood glucose, and oral glucose tolerance test which are relevant for this study have been reported in our previous study.
